# Synthesis of the character impact compound raspberry ketone and additional flavoring phenylbutanoids of biotechnological interest with *Corynebacterium glutamicum*

**DOI:** 10.1186/s12934-020-01351-y

**Published:** 2020-04-21

**Authors:** Lars Milke, Mario Mutz, Jan Marienhagen

**Affiliations:** 1 Institute of Bio- and Geosciences, IBG-1: Biotechnology, Forschungszentrum Jülich GmbH, 52425 Jülich, Germany; 2 Bioeconomy Science Center (BioSC), Forschungszentrum Jülich GmbH, 52425 Jülich, Germany; 3 Institute of Biotechnology, RWTH Aachen University, Worringer Weg 3, 52074 Aachen, Germany

**Keywords:** NADPH-dependent curcumin reductase, Benzalacetone reductase, *Corynebacterium glutamicum*, Raspberry ketone, Metabolic engineering, Character impact compound

## Abstract

**Background:**

The phenylbutanoid 4-(4-hydroxyphenyl)butan-2-one, commonly known as raspberry ketone, is responsible for the typical scent and flavor of ripe raspberries. Chemical production of nature-identical raspberry ketone is well established as this compound is frequently used to flavor food, beverages and perfumes. However, high demand for natural raspberry ketone, but low natural abundance in raspberries, render raspberry ketone one of the most expensive natural flavoring components.

**Results:**

In this study, *Corynebacterium glutamicum* was engineered for the microbial synthesis of the character impact compound raspberry ketone from supplemented *p*-coumaric acid. In this context, the NADPH-dependent curcumin/dihydrocurcumin reductase CurA from *Escherichia coli* was employed to catalyze the final step of raspberry ketone synthesis as it provides a hitherto unknown benzalacetone reductase activity. In combination with a 4-coumarate: CoA ligase from parsley (*Petroselinum crispum*) and a monofunctional benzalacetone synthase from Chinese rhubarb (*Rheum palmatum*), CurA constitutes the synthetic pathway for raspberry ketone synthesis in *C. glutamicum*. The resulting strain accumulated up to 99.8 mg/L (0.61 mM) raspberry ketone. In addition, supplementation of other phenylpropanoids allowed for the synthesis of two other naturally-occurring and flavoring phenylbutanoids, zingerone (70 mg/L, 0.36 mM) and benzylacetone (10.5 mg/L, 0.07 mM).

**Conclusion:**

The aromatic product portfolio of *C. glutamicum* was extended towards the synthesis of the flavoring phenylbutanoids raspberry ketone, zingerone and benzylacetone. Key to success was the identification of CurA from *E. coli* having a benzalacetone reductase activity. We believe, that the constructed *C. glutamicum* strain represents a versatile platform for the production of natural flavoring phenylbutanoids at larger scale.

## Introduction

The phenylbutanoid character impact compound raspberry ketone (4-(4-hydroxyphenyl)butan-2-one, RK) defines the typical scent and taste of raspberries. Thus, it is utilized by food and beverage industries to flavor beverages and foods, e.g. pudding, yogurt or sweets [1, 2]. In addition, its presumed activity as an anti-obesity or skin-whitening agent, drew consumers interest, although a potential toxicity of this compound for humans has not yet been clarified [2–6].

Different strategies can be followed to obtain RK, e.g. extraction from natural plant material or chemical synthesis. Adversely, the natural concentration of RK in raspberries is not only very low (1–4 mg/kg), but also subject to seasonal and regional fluctuations, leading to high product costs of 3000–20,000 US$ per kg of natural, extracted RK [1, 7, 8]. Alternatively, RK can be chemically synthesized, but any RK produced by such processes is only considered as a nature-identical flavoring substance according to EU and US regulations, which no longer meets customers’ demands [9–11]. Contrary to this, RK obtained from microbial synthesis is regarded as natural. Thus, microbial synthesis represents a promising approach for the sustainable production of natural RK. Prerequisite for establishing a microbial RK production process is the functional introduction of the natural biosynthesis pathway from the plant into a heterologous host.

**Fig. 1:**
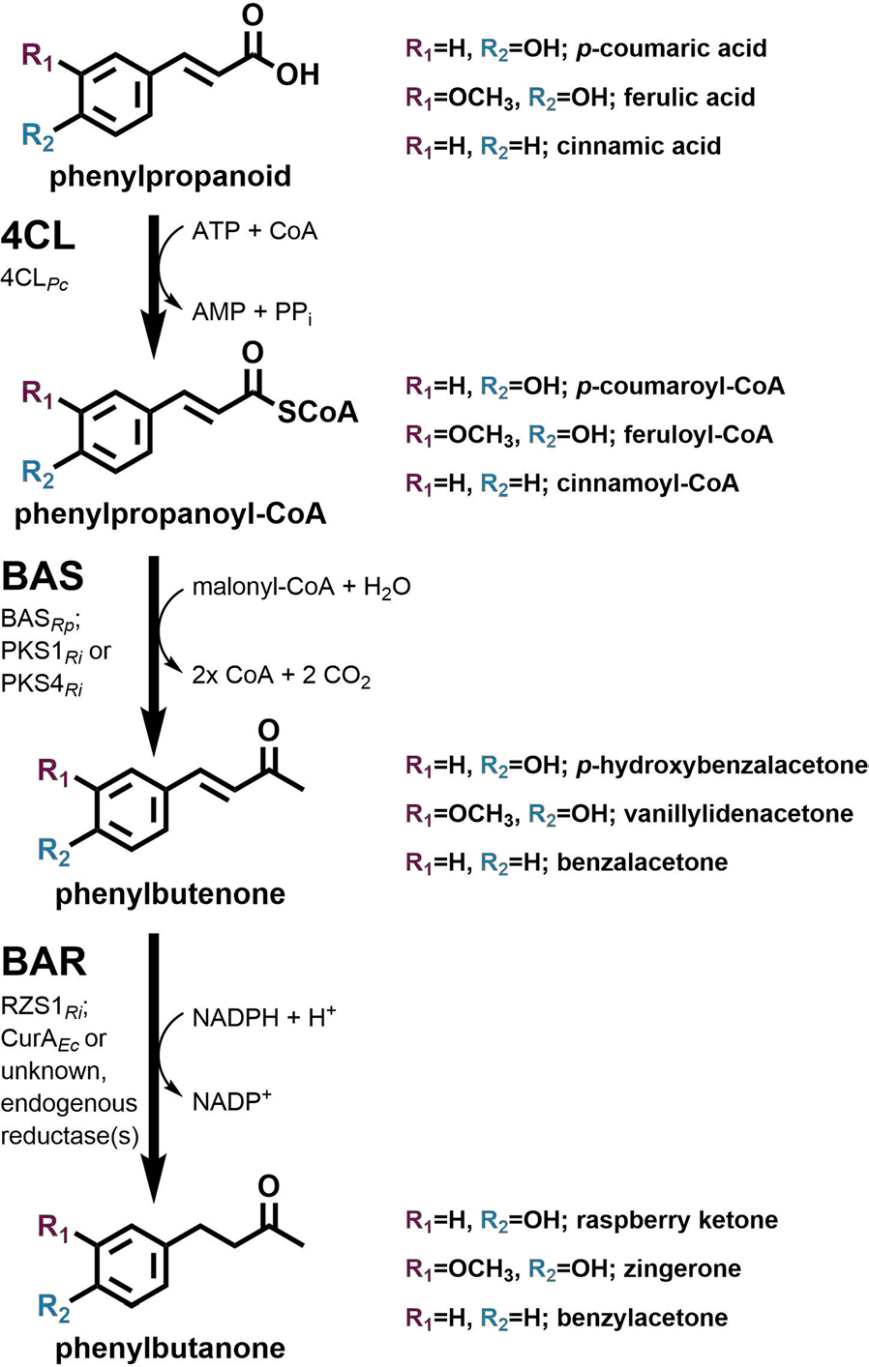
Synthesis of different phenylbutanoids from phenylpropanoids. 4CL: 4-coumarate: CoA ligase, BAS: benzalacetone synthase, BAR: benzalacetone reductase, CoA: coenzyme A. Particular enzymes used in this study are assigned below the respective general enzyme activities. For further information on the individual enzymes used, the reader is referred to the text

In raspberry plants, RK synthesis starts from l-phenylalanine, which is provided by the shikimate pathway [12]. From there, l-phenylalanine is non-oxidatively deaminated by a phenylalanine ammonia lyase (PAL), yielding the phenylpropanoid cinnamic acid, which is subsequently hydroxylated towards *p*-coumaric acid (*p*CA). This compound in turn undergoes CoA-activation catalyzed by a 4-coumarate: CoA ligase (4CL, Fig. 1). The activated thioester is then condensed with one molecule of malonyl-CoA by a benzalacetone synthase (BAS), a type III polyketide synthase (PKS), yielding the diketide intermediate *p*-hydroxybenzalacetone (*p*HBA). Finally, a NADPH-dependent benzalacetone reductase (BAR) reduces *p*HBA to RK.

First studies on microbial RK production from supplemented *p*CA using BAS from Chinese rhubarb (*Rheum palmatum*) and CHS from raspberry (*Rubus idaeus*), respectively, reported product titers below 10 mg/L (0.06 mM) when using *Escherichia coli* or *Saccharomyces cerevisiae* as host strains [1, 13]. Interestingly, both studies relied on endogenous BAR activities by unknown endogenous reductase(s) in the respective host, rendering heterologous expression of a BAR-encoding gene unnecessary. Only recently, synthesis of up to 91 mg/L (0.55 mM) RK was demonstrated using *E. coli* BL21(DE3), which was developed for the expression of *bas* from *R. palmatum* and *rzs1* from raspberry [8, 14]. The latter gene codes for the raspberry ketone/zingerone synthase RZS1 (RZS1_*Ri*_, UniProt ID: G1FCG0), which provides the required BAR activity.

Since various type III PKS-encoding genes of plant origin (encoding for stilbene synthases, chalcone synthases and a pentaketide chromone synthase) have been functionally expressed in *Corynebacterium glutamicum* previously, it is reasonable to assume that this is also true for a type III PKS gene providing BAS activity [15, 16].

In this context, *C. glutamicum* strains have been tailored towards increased malonyl-CoA supply for efficient synthesis of plant polyphenols and polyketides [16–18]. This was necessary, as typically only low intracellular concentrations of the unstable fatty acid precursor malonyl-CoA are maintained in microorganisms as its synthesis is strictly regulated, limiting overall product formation [19]. Although only one molecule of malonyl-CoA is required for the synthesis of one RK molecule, a *C. glutamicum* strain with increased malonyl-CoA availability is predestined for also establishing a heterologous pathway for the synthesis of RK.

In this study, we present the construction of a microbial *C. glutamicum* cell factory for the synthesis of the flavoring phenylbutanoids RK, zingerone and benzylacetone. Additionally, we identified a hitherto unknown BAR activity of the NADPH-dependent curcumin/dihydrocurcumin reductase CurA from *E. coli* allowing for the reduction of diketide intermediates.

## Results

### Cytotoxicity of *p*-hydroxybenzalacetone and raspberry ketone

**Fig. 2:**
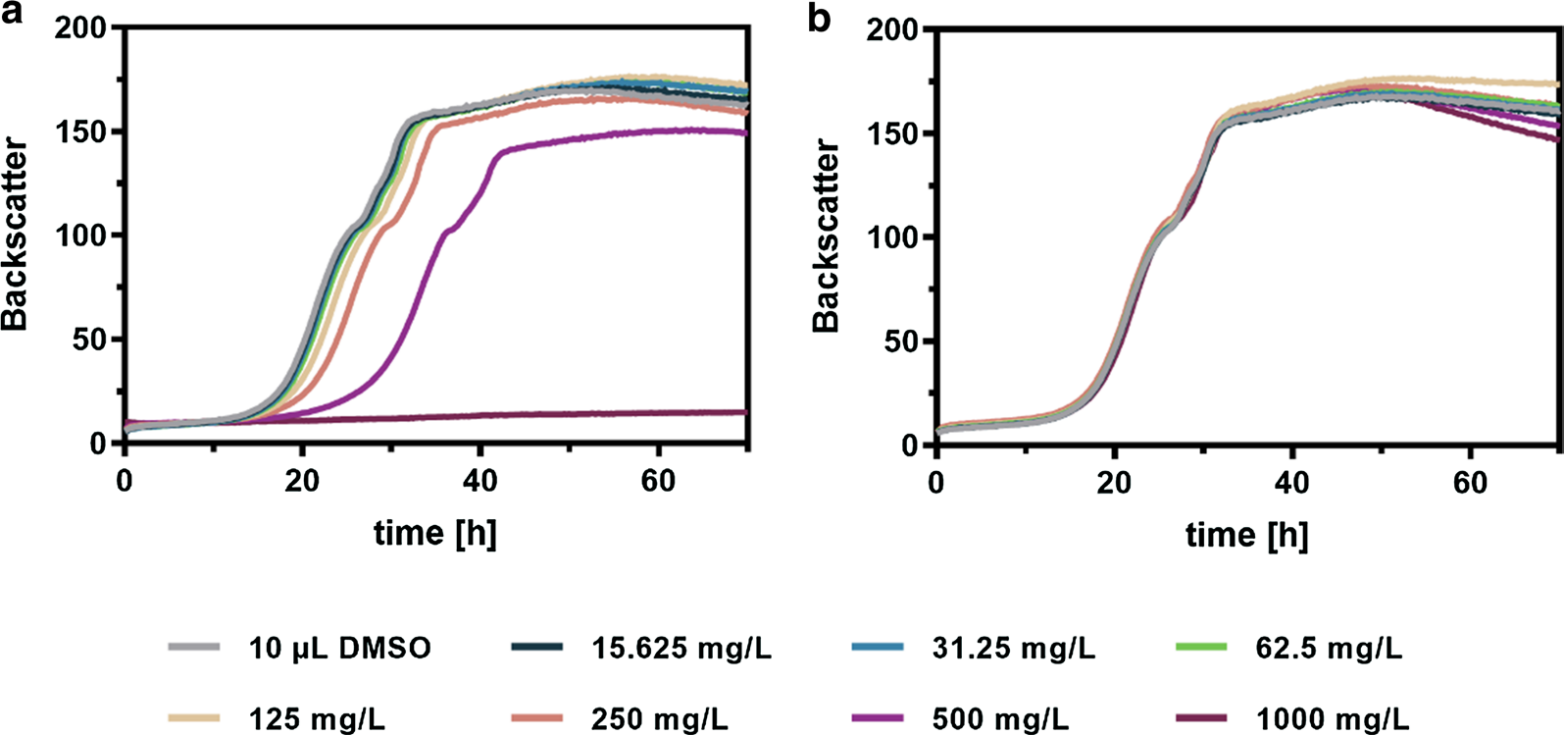
Cytotoxic effects of **a***p*-hydroxybenzalacetone and **b** raspberry ketone on growth of *C. glutamicum*. *C. glutamicum* M-CoA was cultivated in CGXII medium with 4% glucose supplemented with increasing concentrations of either *p*-hydroxybenzalacetone or raspberry ketone dissolved in DMSO using a BioLector microbioreactor. Biomass formation was followed by measuring the backscattered light intensity (gain 10) at a wavelength of 620 nm. The depicted data represent mean values from biological triplicates

In preparation of establishing a heterologous RK biosynthesis pathway from supplemented *p*CA, intermediate (*p*HBA) and product (RK) cytotoxicity on the designated host *C. glutamicum* was investigated. For this purpose, the strain *C. glutamicum* M-CoA, previously constructed for providing increased malonyl-CoA levels [16], was cultivated in CGXII medium with 4% glucose supplemented with different concentrations ranging from 0 to 1 g/L (6.17 mM) *p*HBA and RK (6.1 mM) using the BioLector microbioreactor system (Fig. 2). Concentrations ≥ 125 mg/L (0.77 mM) *p*HBA negatively affected microbial growth up to complete growth inhibition in the presence of 1 g/L (6.17 mM) *p*HBA. Bearing the designated supplementation of 5 mM *p*CA as precursor for RK synthesis in mind, resembling the standard production conditions for the synthesis of *p*CA-derived plant polyphenols using *C. glutamicum*, such toxic concentrations cannot be reached [18]. In contrast, no significant negative impact on growth could be observed upon supplementation of up to 1 g/L (6.1 mM) RK.

Previous studies utilizing either *E. coli* or *S. cerevisiae* for microbial RK synthesis demonstrated that both hosts provide an endogenous BAR activity [1, 13]. Hence, supernatants from the *C. glutamicum* microbioreactor cultivations performed in the context of the *p*HBA cytotoxicity experiments, were analyzed by HPLC for the presence of potentially accumulating RK. Indeed, RK was detected in all samples from cultivations supplemented with ≥ 125 mg/L (0.77 mM) *p*HBA reaching a maximum of 15.4 mg/L (0.094 mM) RK when 500 mg/L (3.09 mM) *p*HBA was present in the microbioreactor cultivations. Interestingly, even though the *C. glutamicum* cells did not grow in the presence of 1 g/L (6.17 mM) *p*HBA, up to 9.3 mg/L (0.057 mM) RK were formed. This particular experiment was repeated without cells to verify that *p*HBA reduction yielding RK is due to the presence of the *C. glutamicum* cells conferring an endogenous BAR activity and not the result of a spontaneous reduction under the selected cultivation conditions in CGXII medium. This control experiment showed that RK formation was only detectable in the presence of *C. glutamicum* cells. Therefore, a yet unknown endogenous BAR activity can also be ascribed to *C. glutamicum*.

### The curcumin reductase CurA from *E. coli* improves the BAR activity in *C. glutamicum*

**Fig. 3:**
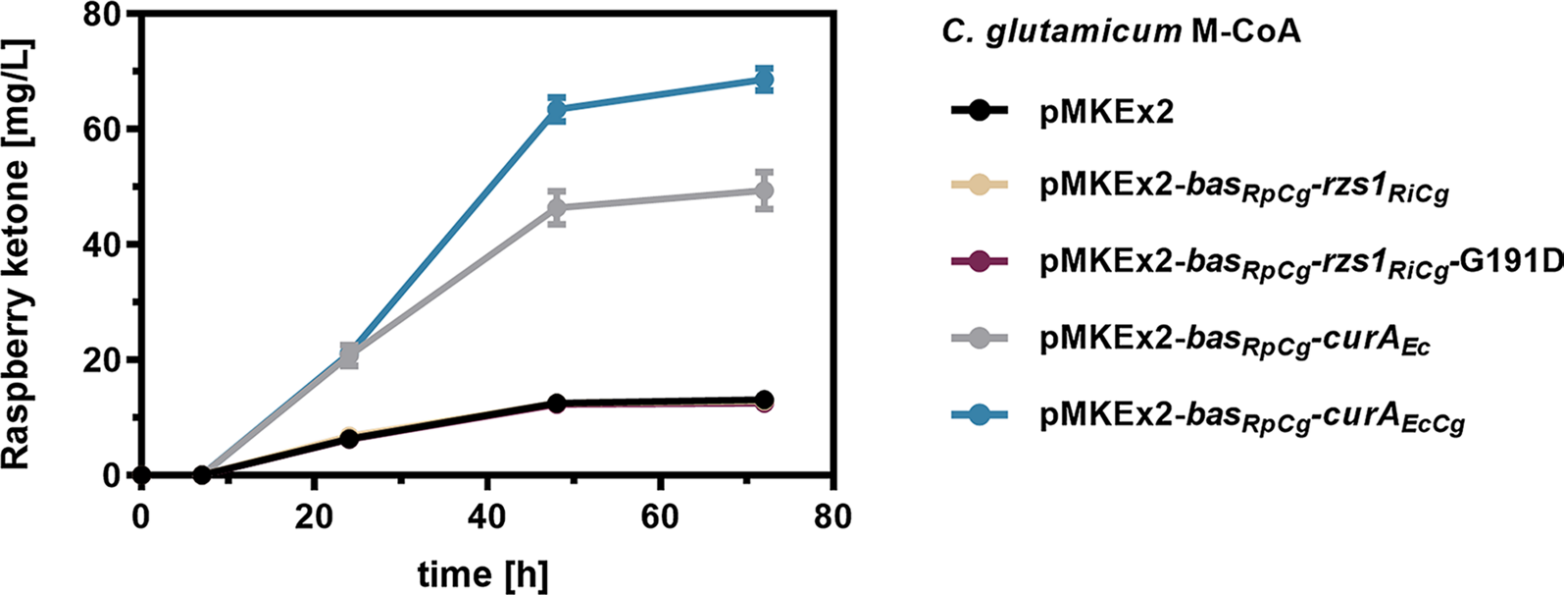
Characterization of different heterologous benzalacetone reductases with regard to their suitability for raspberry ketone synthesis using *C. glutamicum*. Different *C. glutamicum* M-CoA variants harboring one of the indicated expression plasmids were cultivated in 50 mL CGXII medium with 4% glucose and 500 mg/L supplemented *p*-hydroxybenzalacetone in baffled flasks at 30 °C and 130 rpm for 72 h. The depicted data represent mean values with standard deviations from biological triplicates

With the aim to increase the endogenous BAR activity and to establish the full RK pathway in *C. glutamicum*, heterologous genes coding for BAS and BAR enzymes were episomally introduced into this bacterium. For this purpose, a codon-optimized gene variant encoding BAS from *Rheum palmatum* (*bas*_*RpCg*_, UniProt ID: Q94FV7) was combined with a gene for different BAR variants. A codon-optimized gene variant of RZS1_*Ri*_ (*rzs1*_*RiCg*_, UniProt ID: G1FCG0) was used as this particular enzyme has already been successfully applied for the microbial synthesis of RK in *E. coli* [8]. Previously, cofactor specificity in a RZS1_*Ri*_-G191D mutant was reported to be relaxed resulting in the acceptance of NADH as reducing agent [14]. Based on this observation, the same amino acid substitution was also introduced into *rzs1*_*RiCg*_ (*rzs1*_*RiCg*_-G191D). Additionally, available scientific data was analyzed to identify endogenous reductases involved in *p*HBA reduction in *E. coli* and *C. glutamicum*. In case of *E. coli*, the NADPH-dependent curcumin/dihydrocurcumin reductase CurA involved in the degradation of this polyphenol (CurA_*Ec*_, UniProt ID: P76113) was identified as a promising candidate. Its natural substrate curcumin is a dimer of *p*HBA and thus the enzyme might also be active on the monomers (Additional file 1: Figure S1) [20]. Therefore, the native *curA* gene was amplified from the genome of *E. coli* MG1655 (*curA*_*Ec*_) but also ordered as codon-optimized variant (*curA*_*EcCg*_) for a possible application in *C. glutamicum*. To enable IPTG-inducible heterologous gene expression from the strong T7 promotor, the plasmid pMKEx2 was selected [21]. The constructed plasmids were used for the transformation of *C. glutamicum* M-CoA. For evaluation of reductase activity, the generated strains were cultivated for 72 h in 50 mL defined CGXII medium with 4% glucose and 1 mM IPTG supplemented with 500 mg/L (3.09 mM) *p*HBA. Taken samples were extracted with ethyl acetate and analyzed for the synthesis of RK by HPLC (Fig. 3).

Surprisingly, both strains harboring an episomally encoded *rzs1*_*RiCg*_ gene variant did not synthesize more RK from supplemented *p*HBA compared to *C. glutamicum* M-CoA harboring the empty vector (12.9 mg/L, 0.08 mM). This indicates that both *rzs1*_*RiCg*_ variants are not functionally expressed in *C. glutamicum.* However, in presence of the *curA*_*Ec*_, 49.4 mg/L (0.3 mM) RK were synthesized, indicating not only its functional expression in *C. glutamicum* but also the capability of CurA_*Ec*_ to reduce *p*HBA. When utilizing the codon-optimized *curA*_*EcCg*_ gene, RK synthesis was increased further to 68.7 mg/L (0.42 mM). Hence, the pMKEx2-*bas*_*RpCg*_-*curA*_*EcCg*_ plasmid was selected for all subsequent experiments. Noteworthy, at least one additional reductase conferring *p*HBA reducing abilities must be present in *E. coli*, since *E. coli* BL21 strains, previously also utilized for microbial RK synthesis, do not have the *curA* gene [1].

### Increased NADPH availability improves *p*HBA reduction

The observed incomplete conversion of *p*HBA to RK suggested intracellular NADPH supply to be a limiting factor during phenylbutanoid synthesis. With the aim to improve *p*HBA reduction, previously described strategies for increasing NADPH availability were followed [22]. In particular, elimination of the endogenous lactate dehydrogenase activity, especially in combination with the heterologous expression of the transhydrogenase genes *pntAB* from *E. coli,* was shown to contribute to increased NADPH availability in *C. glutamicum*. The constructed strain *C. glutamicum* M-CoA Δ*ldhA* was transformed using the plasmid pMKEx2-*bas*_*RpCg*_-*curA*_*EcCg*_. To evaluate, if the deletion of *ldhA* also increases NADPH availability and thus improves *p*HBA reduction of *C. glutamicum*, strains were cultivated both, absence or presence of pMKEx2-*bas*_*RpCg*_-*curA*_*EcCg*_. Strain cultivation and analysis of taken samples were conducted as described above (Additional file 1: Figure S2). Under standard cultivation conditions, sole *ldhA* deletion did not affect growth, but did also not improve RK synthesis. However, the approach of deleting *ldhA* was not abandoned although no positive effect on absolute RK titers was observed at this stage. Obviously, limited effects on NADPH-dependent *p*HBA reduction generating RK are not surprising when taking into consideration that the abolished lactate-forming reaction is NADH-dependent, consequently increasing NADH availability. For increasing NADPH supply from NADH (NADH + NADP^+^ → NAD^+^ + NADPH), the membrane-bound transhydrogenase PntAB from *E. coli* (UniProt IDs: P07001 and P0AB67) described earlier was tested [22]. *E. coli* harbors two transhydrogenase isoforms. Whereas the energy-dependent PntAB enzyme catalyzes the transfer of a hydride ion from NADH to NADP^+^ under physiological conditions, the energy-independent cytoplasmatic variant UdhA (UniProt ID: P27306) operates in the reverse direction, when an excess of NADPH is present in the cell [23, 24]. However, in principal, both enzymes are capable of catalyzing both reactions.

The expression plasmids pEKEx3-*pntAB*_*Ec*_ and pEKEx3-*udhA*_*EcCg*_, either harboring the native *pntAB* genes from *E. coli* (*pntAB*_*Ec*_) or a codon-optimized *udhA* variant (*udhA*_*EcCg*_), each under control of the *tac* promoter, were constructed. Subsequently, these plasmids were used for transformation of *C. glutamicum* M-CoA Δ*ldhA* harboring pMKEx2-*bas*_*RpCg*_-*curA*_*EcCg*_. The resulting strains were cultivated under the same conditions as described before. As heterologous expression of genes encoding for integral membrane proteins often cause growth defects, multiple IPTG concentrations ranging from 10 to 1000 µM were tested for the heterologous expression of *pntAB*_*Ec*_. These experiments showed that an increasing IPTG concentration was always associated with an increasing growth defect up to a complete arrest of growth (Additional file 1: Figure S3). HPLC analysis indicated a drastically impaired RK synthesis for all IPTG concentrations compared to the reference strain with 1 mM IPTG, rendering the heterologous expression of *pntAB* unsuitable for RK synthesis in *C. glutamicum*.

Contrary, episomal expression of *udhA*_*EcCg*_ barely affected microbial growth but increased RK titers up to 25% (Additional file 1: Figure S4). This indicates that the functional expression of *udhA*_*EcCg*_ in *C. glutamicum* allows for the hydride ion transfer from NADH to NADP^+^. Interestingly, functional expression of *udhA* from *E. coli* in *C. glutamicum* has already been demonstrated earlier, though utilized for the opposite hydride ion transfer [25]. Thus, *C. glutamicum* M-CoA Δ*ldhA* harboring the two expression plasmids pMKEx2-*bas*_*RpCg*_-*curA*_*EcCg*_ and pEKEx3-*udhA*_*EcCg*_ provides the highest BAR activity, resembling a promising candidate for establishing (4CL and) BAS activity to complete the heterologous pathway for RK synthesis from supplemented *p*CA.

### Raspberry ketone synthesis from *p*-coumaric acid

**Fig. 4:**
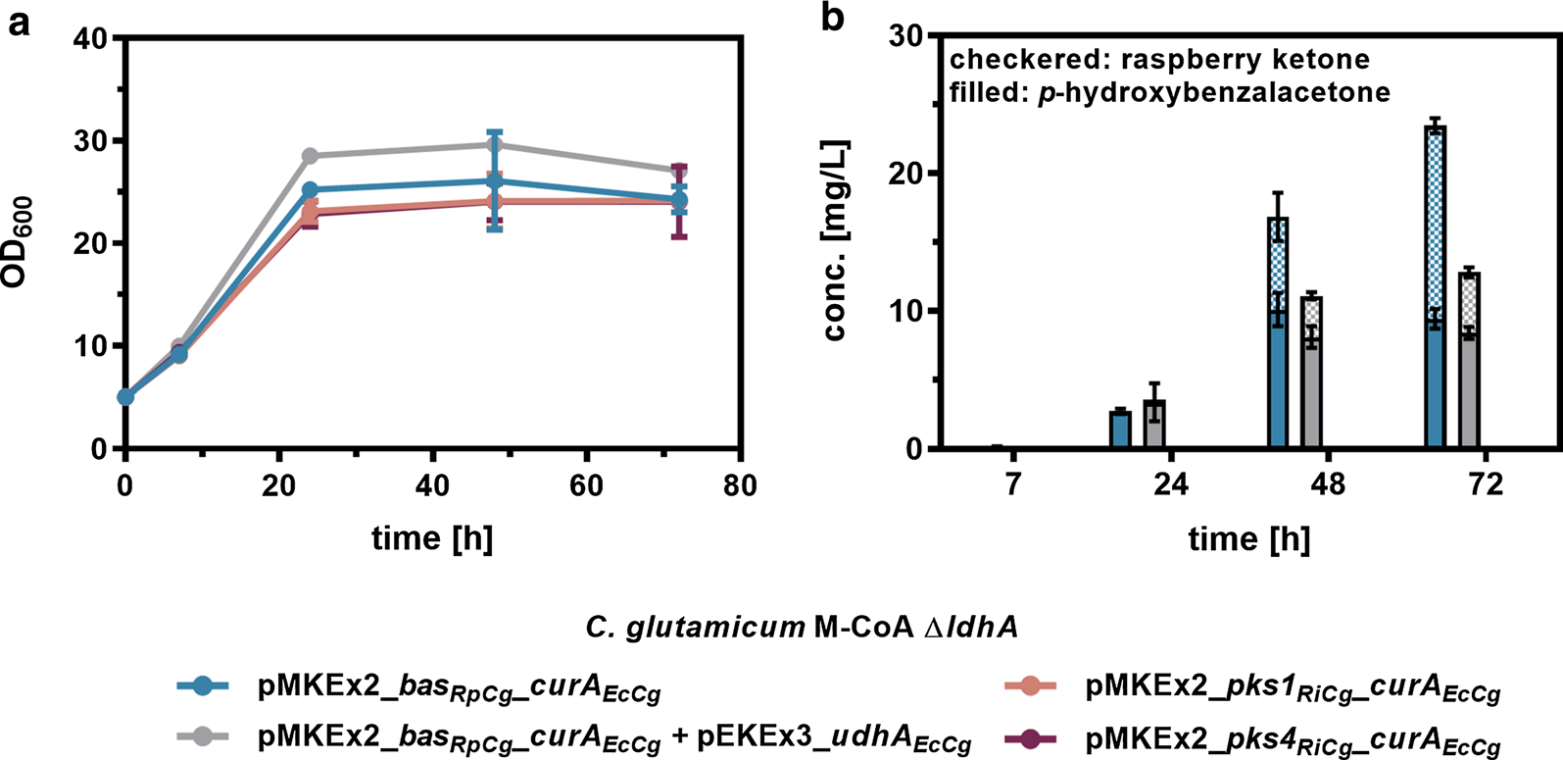
Evaluation of different benzalacetone synthases for raspberry ketone production from *p*-coumaric acid using *C. glutamicum*. *C. glutamicum* M-CoA Δ*ldhA* harboring the indicated expression plasmids was cultivated in 50 mL CGXII medium with 4% glucose and 5 mM *p*-coumaric acid in baffled flasks at 30 °C and 130 rpm for 72 h. **a** Growth and **b***p*-hydroxybenzalacetone- and raspberry ketone synthesis was followed over time. Determined concentrations for *p*-hydroxybenzalacetone and raspberry ketone in cell extracts were stacked for a better visualization. The depicted data represent mean values with standard deviations from biological triplicates

So far, RK synthesis in *C. glutamicum* was only achieved by supplementation of the diketide intermediate *p*HBA. As *C. glutamicum* M-CoA provides increased amounts of malonyl-CoA, synthesis of *p*HBA from *p*CA should be also possible. In addition to BAS from Chinese rhubarb (*R*. *palmatum*), which was shown to feature a novel catalytic mechanism allowing for the sole synthesis of *p*HBA, the bifunctional chalcone synthases PKS1 (UniProt ID: Q9AU11) and PKS4 (UniProt ID: B0LDU5) from raspberry (*R. idaeus*) were tested for *p*HBA synthesis from supplemented *p*CA in *C. glutamicum* [26–29]. Both enzymes were described to have a BAS side activity in addition to their CHS activity. Codon-optimized gene variants *pks1*_*RiCg*_ and *pks4*_*RiCg*_ were used to construct pMKEx2-*pks1*_*RiCg*_-*curA*_*EcCg*_ and pMKEx2-*pks4*_*RrCg*_-*curA*_*EcCg*_. For evaluation of (4CL and) BAS activity, the constructed strains were cultivated and analyzed as described above with supplementation of 5 mM *p*CA instead of 3.09 mM *p*HBA. Stacked concentrations of *p*HBA and RK were used to assess the (4CL and) BAS activity (Fig. 4).

Utilization of the already applied *bas* gene from *R. palmatum* enabled RK synthesis from *p*CA in *C. glutamicum*. After 72 h of cultivation in the absence of *udhA*_*EcCg*_, 14 mg/L (0.09 mM) RK and 9.4 mg/L (0.06 mM) *p*HBA were detected by HPLC. In contrast to the previous experiments, *udhA*_*EcCg*_ expression reduced *p*HBA- and RK synthesis. In addition, neither expression of *pks1* nor *pks4* in combination with *curA* enabled RK synthesis, suggesting that both genes were not functionally expressed in *C. glutamicum.* Since both enzymes are primarily chalcone synthases, it was also tested whether naringenin chalcone, or more precisely, naringenin was formed. However, also no detectable amounts of naringenin were synthesized, indicating that PKS1 and PKS4 might be incorrectly folded in *C. glutamicum*. Previously, N-terminal translational fusion with the maltose binding protein from *E. coli* (MalE_*Ec*_) was demonstrated to efficiently increase functional expression of heterologous plant genes in *C. glutamicum* [30]. To test whether functional expression of *pks* genes could be achieved by mimicking this strategy, *C. glutamicum* strains harboring the plasmids pMKEx2-*malE*_*Ec*_-*pks1*_*RiCg*_-*curA*_*EcCg*_ and pMKEx2-*malE*_*Ec*_-*pks4*_*RiCg*_-*curA*_*EcCg*_ were constructed and cultivated. Although, general applicability of this strategy was indicated by the formation of RK when expressing *malE*_*Ec*_-*pks1*_*RiCg*_ and *malE*_*Ec*_-*pks4*_*RiCg*_, significantly less RK was formed in comparison to BAS_*Rp*_.

Taken together, *C. glutamicum* M-CoA Δ*ldhA* harboring pMKEx2-*bas*_*RpCg*_-*curA*_*EcCg*_ and pEKEx3-*udhA*_*EcCg*_ is regarded as the most suitable strain for the synthesis of RK from supplemented diketide intermediate *p*HBA, whereas additional *udhA*_*EcCg*_ expression was not beneficial for the synthesis from *p*CA.

### Microbial synthesis of zingerone and benzylacetone

**Fig. 5:**
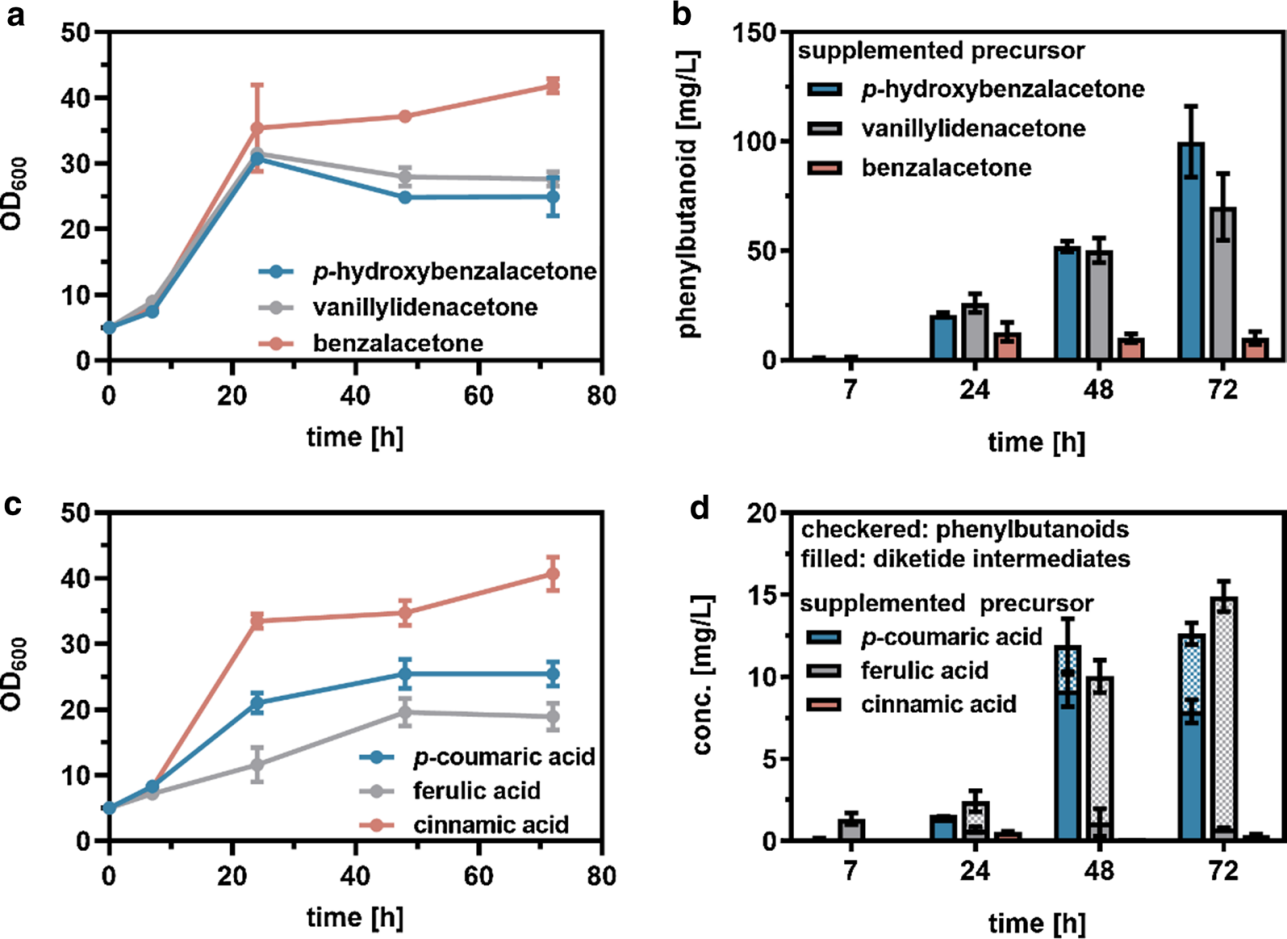
Combinatorial biosynthesis of phenylbutanoids zingerone and benzylacetone from supplemented phenylpropanoid- and diketide precursors using *C. glutamicum* M-CoA Δ*ldhA* pMKEx2-*bas*_*RpCg*_-*curA*_*EcCg*_ pEKEx3-*udhA*_*EcCg*_. Cultivations were performed in 50 mL CGXII medium with 4% glucose and either 3.09 mM diketide or 5 mM phenylpropanoid precursors in baffled flasks at 30 °C and 130 rpm for 72 h. Here, supplementation of *p*-coumaric acid and *p*-hydroxybenzalacetone for the synthesis of RK served as a positive control. **a** Microbial growth and **b** phenylbutanoid synthesis from supplemented diketides. **c** Microbial growth and **d** phenylbutanoid synthesis from supplemented phenylpropanoids. Where appropriate, determined concentrations for diketides (filled bars) and phenylbutanoids (checkered bars) were stacked for a better data visualization. Depicted data represent mean values with standard deviations from biological triplicates

Besides RK, other phenylbutanoids such as the ferulic acid-derived zingerone or the cinnamic acid-derived benzylacetone are of commercial interest as well. Zingerone is regarded as the molecule providing the characteristic flavor of cooked ginger, whereas benzylacetone is described to contribute to the characteristic taste of strawberries and jasmine [31–34]. The respective molecules differ from RK only in their hydroxylation/methoxylation pattern of the aromatic ring (Fig. 1). Thus, it is reasonable to assume that the enzymes of the RK pathway also accept ferulic acid and cinnamic acid (and their derivatives) as substrates, which would offer the opportunity for a combinatorial biosynthesis of zingerone or benzylacetone using the very same *C. glutamicum* strain (Fig. 1). First, confirmation of reductase activity with the respective diketide intermediates of zingerone and benzylacetone synthesis was addressed as formation of the diketide *p*HBA from *p*CA by 4CL_*Pc*_ and BAS_*Rp*_ was rather inefficient and might be even more challenging with alternative phenylpropanoids as substrates. To this end, CGXII medium was supplemented with the respective diketide precursors (3.09 mM) during cultivations of *C. glutamicum* M-CoA Δ*ldhA*, optionally harboring pMKEx2-*bas*_*RpCg*_-*curA*_*EcCg*_. HPLC analysis of extracted samples demonstrated synthesis of 40.2 mg/L (0.21 mM) zingerone upon *curA*_*EcCg*_ expression whereas the synthesis of benzylacetone was unaffected (0.6 mg/L, 0.01 mM) indicating that CurA_*EcCg*_ cannot reduce benzalacetone (Additional file 1: Figure S5). Therefore, benzalacetone appears to be solely reduced by the unknown endogenous reductase activity of *C. glutamicum* yielding benzylacetone. Interestingly, less zingerone (70 mg/L, 0.36 mM) compared to RK (99.8 mg/L, 0.61 mM) was produced from the respective diketide intermediate despite an even higher similarity to the curcumin structure (Fig. 5b).

Nevertheless, reductase activity of the constructed strain *C. glutamicum* M-CoA Δ*ldhA* carrying pMKEx2-*bas*_*RpCg*_-*curA*_*EcCg*_ and pEKEx3-*udhA*_*EcCg*_ was verified for all tested substitution patterns of the aromatic ring.

To evaluate substrate promiscuity of the diketide forming enzymes 4CL_*Pc*_ and BAS_*Rp*_, *C. glutamicum* M-CoA Δ*ldhA* harboring pMKEx2-*bas*_*RpCg*_-*curA*_*EcCg*_ and pEKEx3-*udhA*_*EcCg*_ was cultivated using standard conditions with supplementation of the respective phenylpropanoids (5 mM). Extracted samples were analyzed by HPLC for the presence of respective diketides and ketones (Fig. 5d). After 72 h of cultivation, 7.9 mg/L (0.05 mM) *p*HBA and 4.7 mg/L (0.05 mM) RK were formed from *p*CA. When supplementing either ferulic acid or cinnamic acid, 0.8 mg/L (0.01 mM) vanillylidenacetone and 14.1 mg/L (0.07 mM) zingerone or 0.4 mg/L (0.01 mM) benzalacetone but no detectable benzylacetone was formed, respectively.

In principle, the precursors and intermediates of zingerone and benzylacetone synthesis can be converted also by the heterologous pathway for RK synthesis established in *C. glutamicum*. Nevertheless, benzylacetone could not be produced from cinnamic acid, probably due to the insufficient synthesis of the diketide intermediate benzalacetone. Contrary to previous results obtained from cultivations with supplemented diketide intermediates, the reduction of vanillylidenacetone appears to be more efficient compared to *p*HBA reduction, as almost all vanillylidenacetone synthesized was converted to zingerone.

## Discussion

In this study, we constructed a *C. glutamicum* variant for the microbial synthesis of the flavoring phenylbutanoids RK, zingerone and benzylacetone. Initial cytotoxicity experiments of *p*HBA and RK suggested *C. glutamicum* to be more resistant to these compounds compared to *E. coli* and *S. cerevisiae*. For the latter two microorganisms, half maximal inhibitory concentrations (IC_50_) have been calculated for both *S. cerevisiae* and *E. coli* [1]. Here, concentrations of 100 mg/L or 300 mg/L *p*HBA and 500 mg/L or 900 mg/L were determined for *S. cerevisiae* and *E. coli* to reduce biomass formation by 50%, respectively. Since the calculation of IC_50_ values for *C. glutamicum* would be imprecise due to insufficient data for higher concentrations of both molecules, we cannot provide exact concentrations. Nevertheless, the cytotoxicity experiments allow to consider *C. glutamicum* to be more resistant to both *p*HBA and RK as the IC_50_ concentrations have to be > 500 mg/L and > 1000 mg/L, respectively. More importantly, the constructed strain *C. glutamicum* M-CoA Δ*ldhA* pMKEx2-*bas*_*RpCg*_-*curA*_*EcCg*_ accumulates up to 14 mg/L (0.09 mM) RK from supplemented *p*CA, which is comparable to the product titer determined for a *S. cerevisiae* strain (7.5 mg/L RK (0.05 mM)) [13]. However, synthesis of 91 mg/L (0.55 mM) RK from *p*CA using an engineered *E. coli* BL21(DE3) variant was recently reported [8].

Moreover, a yet unknown substrate promiscuity of the NADPH-dependent curcumin/dihydrocurcumin reductase CurA from *E. coli* MG1655 allowing for the efficient reduction of *p*HBA and vanillylidenacetone, respectively, was identified. Although *E. coli* BL21 has been previously reported to possess an endogenous BAR activity, this activity presumably cannot be traced back to CurA as this particular gene is not present in the utilized strain background [1]. Thus, it is likely that at least one additional reductase also features BAR activity in *E. coli* BL21. We presumed a possible enzymatic activity of CurA with *p*HBA due to structural similarities with its natural substrate curcumin (Additional file 1: Figure S1). As the additional methoxy group of vanillylidenacetone increases structural similarity to curcumin even more, a more efficient conversion to the corresponding ketone compared to the RK branch was expected prior to the conducted experiments. Indeed, when producing flavoring phenylbutanoids from supplemented phenylpropanoids, an almost complete conversion of vanillylidenacetone to zingerone was observed, whereas *p*HBA reduction was less efficient.

Furthermore, a hitherto unknown endogenous BAR activity must be also present in *C. glutamicum* as demonstrated by the reduction of the three tested diketide intermediates in the absence of *curA*_*EcCg*_. Apart from *E. coli* and *C. glutamicum*, such an activity has already been described for *S. cerevisiae* [13].

To increase NADPH supply for the efficient reduction of diketide intermediates, heterologous expression of genes encoding the membrane-bound as well as the cytoplasmatic transhydrogenases from *E. coli* was evaluated. Despite already being used to increase NADPH availability in an isobutanol producing *C. glutamicum* variant, the membrane-bound transhydrogenase PntAB turned out to be unsuitable for RK synthesis with the same bacterium [22]. With increasing induction strength, a severe growth defect in *C. glutamicum* was observed upon *pntAB* expression. This might indicate cytotoxic effects of the transhydrogenase itself, but also the absence of chaperones supporting folding or an altered membrane composition could be the cause [35]. Contrary, the cytoplasmatic transhydrogenase UdhA was beneficial for RK synthesis resembling a promising alternative to the membrane-bound PntAB. Despite being rather involved in the energy-independent hydride ion transfer from NADPH to NAD^+^ in vivo, UdhA can still catalyze the transfer in the reverse direction [23]. Nevertheless, the equilibrium of the transhydrogenation reaction could be shifted towards NADPH, when NADPH is constantly withdrawn by the reduction of *p*HBA to RK. Furthermore, deletion of *ldhA* increases NADH availability, which in turn also shifts the equilibrium of the transhydrogenase reaction towards NADPH. Further strategies for increased NADPH supply in *C. glutamicum* involve altering the coenzyme specificity of the NAD^+^-dependent glyceraldehyde 3-phophate dehydrogenase (GAPDH) to NADP^+^, which was done in the context of l-lysine production with *C. glutamicum* [36]. It should be noted, that an imbalanced NADH/NADPH distribution could perturb the cellular metabolism and might even inhibit cellular growth or glucose consumption [36].

Interestingly, expression of *udhA*_*EcCg*_ appears to be disadvantageous for the synthesis of *p*HBA and RK from *p*CA. NADPH availability could not be limiting for the small amounts of *p*HBA produced from *p*CA, so that the described positive effect of additional NADPH supply only become significant at higher *p*HBA concentrations. The reduced cumulated titer of *p*HBA and RK might be due to the increased metabolic burden of the cell due to maintenance of the pEKEx3-*udhA*_*EcCg*_ plasmid (requiring supplementation of a second antibiotic and expression of an additional antibiotic resistance gene) [37, 38].

## Conclusion

In the present work, we extended the product portfolio of *C. glutamicum* towards flavoring phenylbutanoids. We identified an endogenous BAR activity of *C. glutamicum* and a yet unknown substrate promiscuity of CurA from *E. coli* that turned out to be a promising BAR. Moreover, the cytoplasmatic transhydrogenase UdhA from *E. coli* allowed for increased NADPH supply and ultimately improved RK synthesis. Taken together, the constructed strain *C. glutamicum* M-CoA Δ*ldhA* harboring pMKEx2-*bas*_*RpCg*_-*curA*_*EcCg*_ and pEKEx3-*udhA*_*EcCg*_ represents a versatile host for the synthesis of up to 99.8 mg/L (0.61 mM) RK, 70 mg/L (0.36 mM) zingerone and 10.5 mg/L (0.07 mM) benzylacetone.

## Materials and methods

### Bacterial strains, plasmids, media and growth conditions

All bacterial strains and plasmids with their respective characteristics used in this study are listed in Table 1. *C. glutamicum* strains were routinely cultivated aerobically at 30 °C in brain heart infusion (BHI) medium (Difco Laboratories, Detroit, USA) or defined CGXII medium with 4% (w/v) glucose as sole carbon and energy source [39]. *E. coli* DH5α, used solely for plasmid constructions, was cultivated in LB medium at 37 °C [40]. Where appropriate, kanamycin (*E. coli* 50 µg/mL, *C. glutamicum* 25 µg/mL) and/or spectinomycin (100 µg/mL for *E. coli* and *C. glutamicum*) was added to the respective medium. Bacterial growth was followed by measuring the optical density at 600 nm (OD_600_).

**Table 1:** Strains and plasmids used in this study

Strain or plasmid	Characteristics	Source
*C. glutamicum* strains		
DelAro^4^-*4cl*_*Pc*_ C5 mu*fasO*_*BCD1*_ P_O6_-*iolT1* Δ*pyc* (M-CoA)	*C. glutamicum* derivative with in-frame deletions of cg0344-47, cg0503 cg2625-40 and cg1226; harboring a chromosomally encoded codon-optimized *4cl*_*Pc*_ gene coding for 4-coumarate:CoA ligase from *P. crispum* under control of the T7 promoter and replacement of the native *gltA* promotor with the *dapA* promotor variant C5, mutated *fasO* binding sites upstream of *accBC* and *accD1*, two nucleotide exchanges in the *iolT1* promotor and in-frame deletion of *pyc*	[16]
M-CoA Δ*ldhA*	*C. glutamicum* M-CoA derivative with in-frame deletion of *ldhA*	This work
*E. coli* strains		
DH5α	F^–^Φ80*lacZ*ΔM15 Δ(*lacZYA*-*argF*)U169 *recA1 endA1 hsdR*17 (r_K_^–^,m_K_^+^) *phoA supE*44 λ^–^ *thi*-*1 gyrA*96 *relA1*	Invitrogen (Karlsruhe, Germany)
Plasmids		
pK19*mobsacB*-Δ*ldhA*	Vector for in-frame deletion of *ldhA*	[46]
pMKEx2	*kan*^r^; *E. coli*-*C. glutamicum* shuttle vector (*lacI*, P_T7_, *lacO1*, pHM1519 ori_*Cg*_; pACYC177 ori_*Ec*_)	[21]
pMKEx2-*bas*_*RpCg*_-*rzs1*_*RiCg*_	*kan*^r^; pMKEx2 derivative containing codon-optimized genes encoding benzalacetone synthase from *R. palmatum* (*bas*_*RpCg*_) and NADPH-dependent raspberry ketone/zingerone reductase from *R. idaeus* (*rzs1*_*RiCg*_)	This work
pMKEx2-*bas*_*RpCg*_-*rzs1*_*RiCg*_-G191D	*kan*^r^; pMKEx2-*bas*_*RpCg*_-*rzs1*_*RiCg*_ derivative with mutations in the rzs1_*RiCg*_ nucleotide sequence leading to amino acid substitution G191D in RZS1_*Ri*_.	This work
pMKEx2-*bas*_*RpCg*_-*curA*_*Ec*_	*kan*^r^; pMKEx2 derivative containing a codon-optimized gene encoding benzalacetone synthase from *R. palmatum* (*bas*_*RpCg*_) and the native gene encoding NADPH-dependent curcumin/dihydrocurcumin reductase CurA from *E.* *coli* (*curA*_Ec_)	This work
pMKEx2-*bas*_*RpCg*_-*curA*_*EcCg*_	*kan*^r^; pMKEx2 derivative containing codon-optimized genes encoding benzalacetone synthase from *R. palmatum* (*bas*_*RpCg*_) and NADPH-dependent curcumin/dihydrocurcumin reductase CurA from *E.* *coli* (*curA*_*EcCg*_)	This work
pMKEx2-*pks1*_*RiCg*_-*curA*_*EcCg*_	*kan*^r^; pMKEx2 derivative containing codon-optimized genes encoding PKS1 from *R. idaeus* (*pks1*_*RiCg*_) and NADPH-dependent curcumin/dihydrocurcumin reductase CurA from *E.* *coli* (*curA*_*EcCg*_)	This work
pMKEx2-*pks4*_*RiCg*_-*curA*_*EcCg*_	*kan*^r^; pMKEx2 derivative containing codon-optimized genes encoding PKS4 from *R. idaeus* (*pks4*_*RiCg*_) and NADPH-dependent curcumin/dihydrocurcumin reductase CurA from *E.* *coli* (*curA*_*EcCg*_)	This work
pMKEx2-*malE*_*Ec*_-*pks1*_*RiCg*_-*curA*_*EcCg*_	*kan*^r^; pMKEx2 derivative containing the native *malE* gene from *E. coli* fused to the codon-optimized gene encoding PKS1 from *R. idaeus* (*malE*_*Ec*_-*pks1*_*RiCg*_) and a codon-optimized gene encoding NADPH-dependent curcumin/dihydrocurcumin reductase CurA from *E.* *coli* (*curA*_*EcCg*_)	This work
pMKEx2-*malE*_*Ec*_-*pks4*_*RiCg*_-*curA*_*EcCg*_	*kan*^r^; pMKEx2 derivative containing the native *malE* gene from *E. coli* fused to the codon-optimized gene encoding PKS4 from *R. idaeus* (*malE*_*Ec*_-*pks4*_*RiCg*_) and a codon-optimized gene encoding NADPH-dependent curcumin/dihydrocurcumin reductase CurA from *E.* *coli* (*curA*_*EcCg*_)	This work
pEKEx3	*spec*^r^; *E. coli*-*C. glutamicum* shuttle vector (*lacI*,P_tac_, *lacO1*, pBL1ori_*Cg*_; pUCori_*Ec*_)	[47]
pEKEx3-*pntAB*_*Ec*_	*spec*^r^; pEKEx3 derivative containing native *pntAB* genes from *E. coli* (*pntAB*_*Ec*_) encoding a membrane-bound transhydrogenase	This work
pEKEx3-*udhA*_*EcCg*_	*spec*^*r*^; pEKEx3 derivative containing codon-optimized *udhA* gene variant from *E. coli* (*udhA*_*EcCg*_) encoding a cytoplasmatic transhydrogenase	This work
pEKEx3-*malE*_*Ec*_-*omt*_*Vv*_	*spec*^*r*^; pEKEx3 derivative containing *malE* gene from *E. coli* (*malE*_*Ec*_) fused to the codon-optimized gene coding for resveratrol-di-*O*-methyltransferase from *V. vinifera* (*omt*_*Vv*_)	[30]

To cultivate *C. glutamicum*, a test tube with 5 mL BHI medium was inoculated with a single colony from an agar plate and grown for 6–8 h on a rotary shaker at 170 rpm (first preculture). This first preculture was used to inoculate 50 mL of defined CGXII medium with 4% (w/v) glucose in a 500 mL baffled Erlenmeyer flask (second preculture). The second preculture was cultivated overnight on a rotary shaker at 130 rpm. The main culture was subsequently inoculated from the second preculture to the indicated OD_600_ in defined CGXII medium with 4% (w/v) glucose. For microbial synthesis of phenylbutanoids, the main culture was inoculated to an OD_600_ of 5 in defined CGXII medium with 4% glucose and heterologous gene expression was induced 90 min after inoculation using 1 mM IPTG. 1 mL of the culture broth was sampled at defined time points and stored at − 20 °C until ethyl acetate extraction and HPLC analysis.

For evaluating cytotoxicity of *p*HBA and RK, *C. glutamicum* M-CoA was cultivated at 30 °C, 900 rpm and a humidity of 85% in 48-well Flowerplates containing 800 µL CGXII medium with 4% (w/v) glucose inoculated to an OD_600_ of 1, using the BioLector microbioreactor (m2p-labs, Baesweiler, Germany). Increasing concentrations of both *p*HBA and RK (final concentrations 0, 15.625, 31.25, 62.5, 125, 250, 500 and 1000 mg/L), dissolved in 10 µL DMSO were added. Online measurement of the backscattered light intensity (620 nm, gain 10) was used for evaluation of cellular growth. To estimate IC_50_ values, obtained backscattered light intensities after 72 h were plotted against the respective *p*HBA- and RK concentrations and subsequently analyzed using the GraphPad Prism 8.1.2 software (San Diego, CA, USA). The nonlinear regression *[inhibitor] vs. response* − *Variable slope (four parameters)* with the following specifications was used: top = 165, bottom = 15, as well as IC_50_ > 0. The values for top and bottom correspond to the mean values for the determined backscatter values after 72 h in the absence of *p*HBA or RK, or the average value for the backscatter after 72 h in the presence of 1000 mg/L (6.17 mM) *p*HBA.

### Plasmid and strain construction

Standard protocols of molecular cloning, such as PCR, restriction and ligation of DNA were carried out for recombinant DNA work [41]. All enzymes were obtained from Thermo Fisher Scientific (Schwerte, Germany). Codon-optimized synthetic genes for *C. glutamicum* ATCC13032 were obtained from Thermo Fisher Scientific (formerly GeneArt, Darmstadt, Germany). Genes and chromosomal fragments were amplified by PCR from synthetic genes or genomic *E. coli* DNA as template using the listed oligonucleotides (Additional file 1: Table S1). PCR products were subsequently used to clone genes and genomic fragments into plasmid vectors using Gibson assembly [42]. In-frame gene deletions in the *C. glutamicum* genome were performed using the pK19*mobsacB* system by a two-step homologous recombination method described previously [43, 44]. Integrity of all constructed plasmids was verified by colony PCR, restriction analysis, and DNA sequencing at Eurofins MWG Operon (Ebersberg, Germany) Techniques specific for *C. glutamicum*, e.g. electroporation of cells, were performed as described previously [45].

### Ethyl acetate extraction and HPLC quantification

Phenylbutanoids and pathway intermediates were extracted from cultivation broth for subsequent HPLC analysis by mixing 1 mL of the culture broth with 1 mL ethyl acetate and vigorous shaking (1400 rpm, 10 min, 20 °C) in a Thermomixer (Eppendorf, Hamburg, Germany). The suspension was centrifuged for 5 min at 13,000 rpm and the upper ethyl acetate layer (800 µL) was transferred to an organic solvent resistant deep-well plate (Eppendorf, Hamburg, Germany). After evaporation to dryness, extracts were resuspended in the same volume of acetonitrile and subsequently used for HPLC analysis.

Metabolites were quantified using an Agilent high-performance liquid chromatography (HPLC) 1260 Infinity System equipped with a 1260 Infinity DAD (Agilent Technologies, Waldbronn, Germany). Authentic standards of benzalacetone, benzylacetone, cinnamic acid, ferulic acid, *p*-coumaric acid, vanillylidenacetone and zingerone were purchased from Sigma-Aldrich (Taufkirchen, Germany), *p*-hydroxybenzalacetone was obtained from Alfa Aesar (Kandel, Germany) and raspberry ketone from Acros Organics (Geel, Belgium). LC separation was carried out with an InfinityLab Poroshell 120 2.7 µm EC-C_18_ column (3.0 × 150 mm; Agilent Technologies, Waldbronn, Germany) at 50 °C. For elution, 0.1% acetic acid (solvent A) and acetonitrile supplemented with 0.1% acetic acid (solvent B) were applied as the mobile phases at a flow rate of 0.7 mL/min. Depending on the analyte, a different elution gradient was used, where the amount of solvent B was increased stepwise. *Raspberry ketone*: minute 0–10: 10%, minute 10–11: 10–90%, minute 11–13: 90%, minute 13–15: 90–10% and minute 15–17: 10%. Absorption was determined at 275 nm (raspberry ketone), 310 nm (*p*-coumaric acid) and 320 nm (*p*-hydroxybenzalacetone). Z*ingerone*: minute 0–15: 10%, minute 15–16: 10–90%, minute 16–18: 90%, minute 18–20: 90–10% and minute 20–22: 10%. Absorption was determined at 275 nm (zingerone) and 320 nm (ferulic acid and vanillylidenacetone). *Benzylacetone*: minute 0–13: 10–50%, minute 13–15: 50%, minute 15–17: 50–10% and minute 17–19: 10%. Absorption was determined at 260 nm (benzylacetone) and 320 nm (cinnamic acid and benzalacetone). Area values of integrated signals were linear up to metabolite concentrations of at least 83.3 mg/L.

## Supplementary information

**Additional file 1.** Additional information containing a list of oligonucleotides used in this study, the chemical structure of curcumin with highlighted *p*HBA structure and additional cultivation results.

## Data Availability

All data generated or analyzed during this study are included in this published article and its additional files.
