# Supplementary material for: Synthesis of the character impact compound raspberry ketone and additional flavoring phenylbutanoids of biotechnological interest with Corynebacterium glutamicum
Source: Microb Cell Fact. 2020 Apr 21;19:92. doi: 10.1186/s12934-020-01351-y (PMC7175512; doi:10.1186/s12934-020-01351-y)
Supplement: Supplementary file 1 — Additional file 1. Additional information containing a list of oligonucleotides used in this study, the chemical structure of curcumin with highlighted pHBA structure and additional cultivation results. [file 12934_2020_1351_MOESM1_ESM.docx]

**Synthesis of the character impact compound raspberry ketone and additional flavoring phenylbutanoids of biotechnological interest with *Corynebacterium glutamicum***

**Lars Milke^1^, Mario Mutz^1^, Jan Marienhagen^1,2,3 *^**

**^1^**Institute of Bio- and Geosciences, IBG-1: Biotechnology, Forschungszentrum Jülich, D-52425 Jülich, Germany

**^2^**Bioeconomy Science Center (BioSC), Forschungszentrum Jülich GmbH, D-52425 Jülich, Germany

**^3^**Institute of Biotechnology, RWTH Aachen University, Worringer Weg 3, D-52074 Aachen, Germany

e-mail / ORCID ID:

Lars Milke: l.milke@fz-juelich.de 0000-0001-9151-1065

Mario Mutz: m.mutz@fz-juelich.de 0000-0003-1716-6931

^*^ Corresponding author:

Prof. Dr. Jan Marienhagen, phone: +49 2461 61 2843, e-mail: [j.marienhagen@fz-juelich.de](mailto:j.marienhagen@fz-juelich.de)
ORCID ID: 0000-0001-5513-3730

Keywords: NADPH-dependent curcumin reductase, benzalacetone reductase, *Corynebacterium glutamicum*, raspberry ketone, metabolic engineering, character impact compound.

**Table S1: Oligonucleotides used in this study.**

| **Primer** | **Sequence (5'🡪3')** |
| --- | --- |
| bas_RpCg_-s | ATTGTGAGCGGATAACAATTCCCCTCTAGA**AAGGAGG**TCGAAGATGGCAACCGAAGAAATG |
| bas_RpCg_-as | CTCCTTTAGCACCATGGTTAGGAGATCACTGGCAC |
| rzs1_RiCg_-s | AGTGATCTCCTAACCATGGTGCTA**AAGGAGG**TCGAAGATGGCATCCGGTGGCGAA |
| rzs1_RiCg_-as | TCTGCGGCCGCGTCGACTTGTACAGGATCCTTATTCACGGGACACCACCAC |
| rzs1_RiCg_-G191D-s | CCTTTTCTTTGGAATCTGCGGAGCCCACCACGTA |
| rzs1_RiCg_-G191D-as | GGTGGGCTCCGCAGATTCCAAAGAAAAGGTGGATC |
| curA_Ec_-s | CGTGCCAGTGATCTCCTAACCATGGTGCTA**AAGGAGG**TCGAAGATGGGGCAACAAAAGCAG |
| curA_Ec_-as | GGCCGCGTCGACTTGTACAGGATCCTTAATCATCACCCGCCAC |
| curA_EcCg_-s | GATCTCCTAACCATGGTGCTA**AAGGAGG**TCGAAGATGGGCCAGCAGAAGCAG |
| curA_EcCg_-as | GGCCGCGTCGACTTGTACAGGATCCTTAATCGTCGCCAGCCAC |
| pntA_Ec_-s | CCTGCAGGTCGACTCTAGAGGATCC**AAGGAGG**TCATATCATGGAAGGGAATATCATGC |
| pntA_Ec_-as | ACGATTCCTCCTTGTACATTCACGGCCAGATTTAATTTTTGCGGAACATTTTC |
| pntB_Ec_-s | ATCTGGCCGTGAATGTAC**AAGGAGG**AATCGTATGTCTGGAGGATTAGTTACAGCT |
| pntB_Ec_-as | CTGTAAAACGACGGCCAGTGAATTCTTACAGAGCTTTCAGGATTG |
| udhA_EcCg_-s | CCTGCAGGTCGACTCTAGAGGATCC**AAGGAGG**TCATATCATGGGCCTGGTGAAGCAG |
| udhA_EcCg_-as | CTGTAAAACGACGGCCAGTGAATTCTTAGATATCTTCGATCAGGTGTGC |
| pks1_RiCg_-s | GAGCGGATAACAATTCCCCTCTAGA**AAGGAGG**TCGAAGATGGTGACCGTGGATGAAG |
| pks1_RiCg_-as | CTTCGACCTCCTTTAGCACCATGGTTAGGTGGATGCTGCCAC |
| pks4_RiCg_-s | GAGCGGATAACAATTCCCCTCTAGA**AAGGAGG**TCGAAGATGGTGACCGTGGAAGAAG |
| pks4_RiCg_-as | CTTCGACCTCCTTTAGCACCATGGTTACACCAGGGAGAACAG |
| malE_Ec_-s | GAGCGGATAACAATTCCCCTCTAGA**AAGGAGG**TCGAAGATGAAAACTGAAGAAGGTAAACTGGTAATCTG |
| malE_Ec_-as | CCACGGTCACGCCGGAACCGGAAGAGGA |
| malE_Ec_-pks1_RiCg_-s | CGGTTCCGGCGTGACCGTGGAAGAAGTG |
| malE_Ec_-pks4_RiCg_-s | CGGTTCCGGCGTGACCGTGGATGAAGTG |
| chk-pMKEx2-s | CCCTCAAGACCCGTTTAGAGGC |
| chk-pMKEx2-as | TTAATACGACTCACTATAGGGGAATTGTGAGC |
| chk-ldhA-s | GTGCGATGCCTGATCAATCCCACAACCG |
| chk-ldhA-as | GGTTTCATCGTCGTGTGCACAGTTG |
| *C. glutamicum* ribosome binding sites are highlighted in bold, relevant restriction sites are underlined. | |


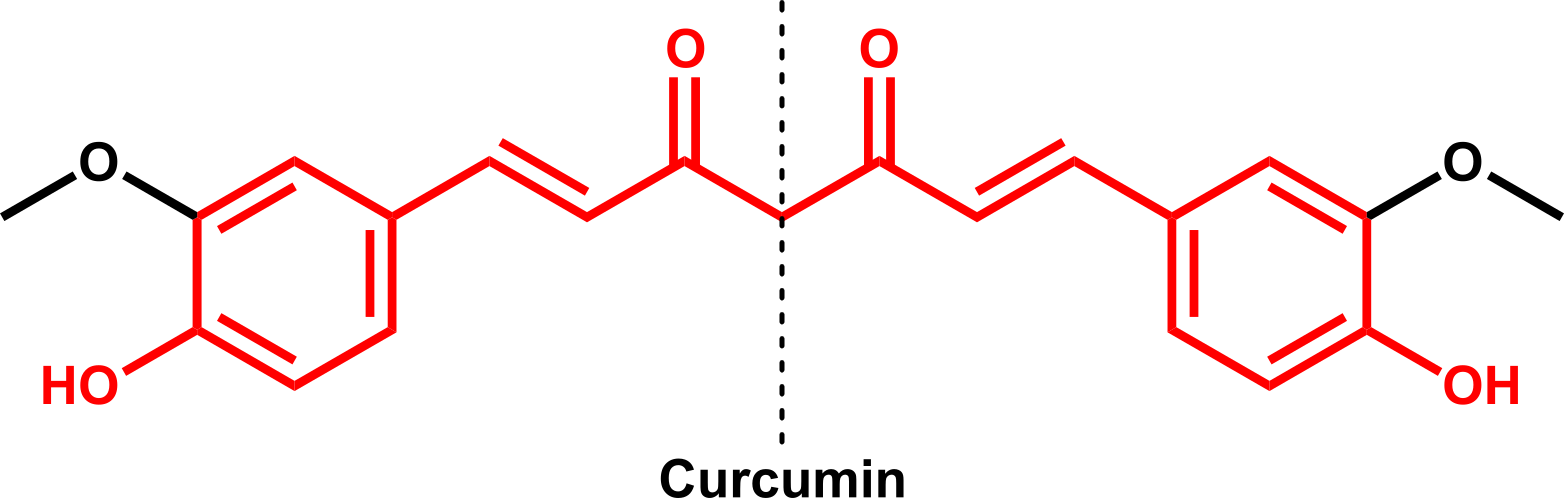


**Figure S1: Chemical structure of curcumin.** The structure of *p*-hydroxybenzalacetone can be found twice in the curcumin structure as highlighted by the red coloration and dashed line.


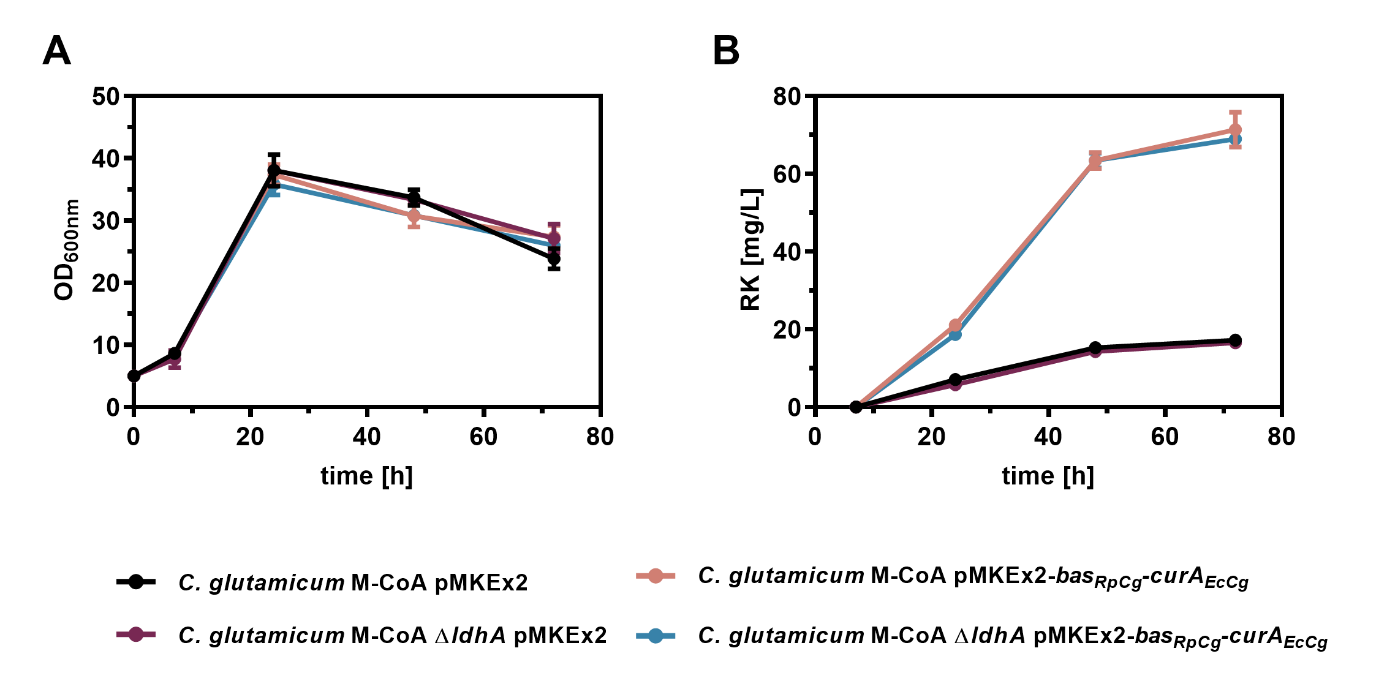


**Figure S2: Effect of *ldhA* deletion on microbial (A) growth of *C. glutamicum* and (B) benzalacetone reductase activity.** Indicated strains were cultivated for 72 hours in 50 ml defined CGXII medium with 4 % glucose and 1 mM IPTG supplemented with 500 mg/L (3.09 mM) *p*HBA. Taken samples were extracted with ethyl acetate and analyzed for the synthesis of RK by HPLC. All data presented are mean values including standard deviations from biological triplicates.


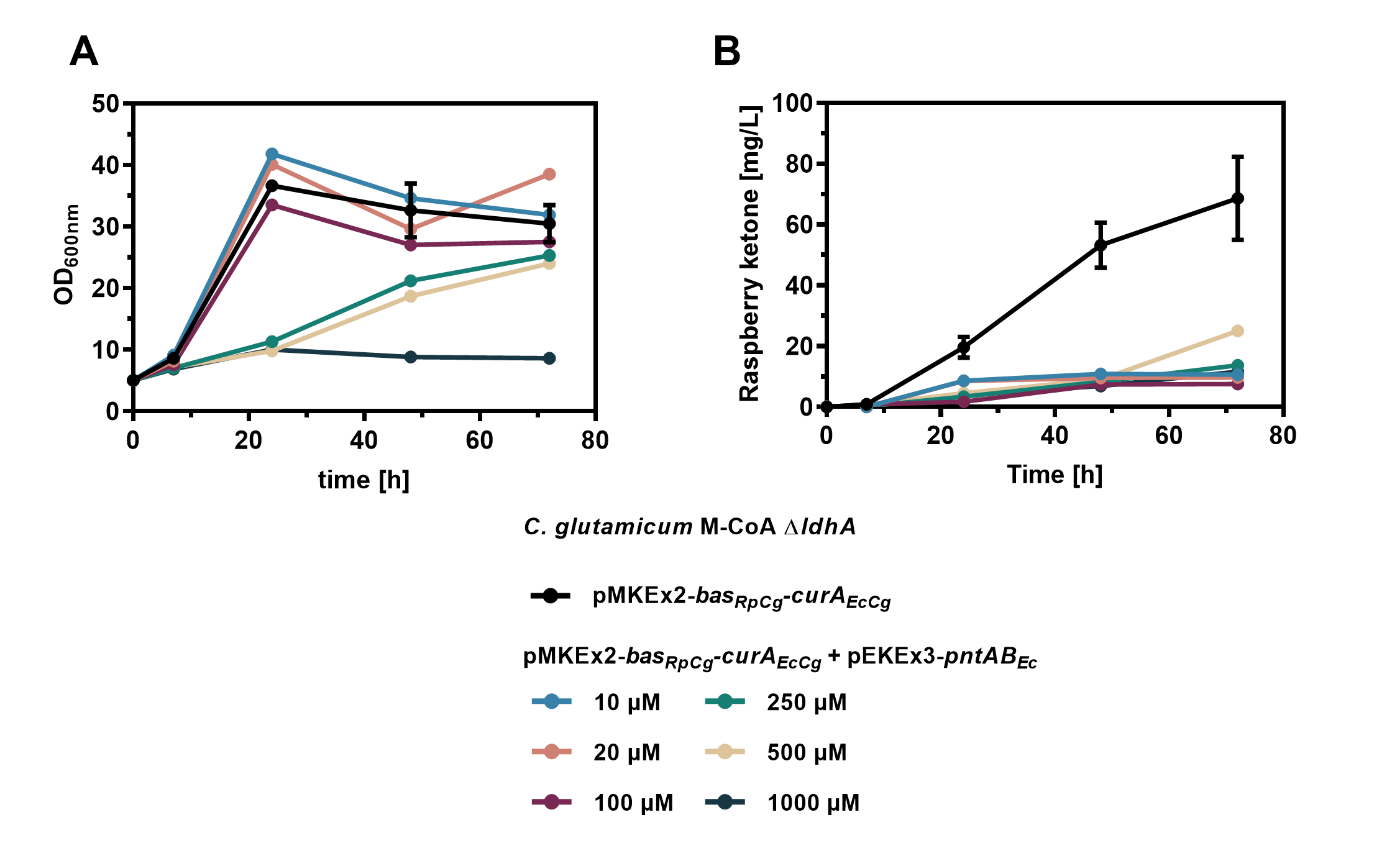


**Figure S3: Effect of heterologous expression of *pntAB_EC_* encoding the membrane bound transhydrogenase from *E. coli* on (A) growth of *C. glutamicum* and (B) raspberry ketone synthesis.** *C. glutamicum* M-CoA Δ*ldhA* harboring the indicated expression plasmids was cultivated in 50 mL CGXII medium with 4 % glucose and 500 mg/L supplemented *p*-hydroxybenzalacetone in baffled flasks at 30 °C and 130 rpm for 72 h. The depicted data for the strain solely harboring pMKEx2-*bas_RpCg_*-*curA_EcCg_* (black) represents mean values with standard deviations from biological triplicates. The coloured data points indicating the varying IPTG concentrations (10 - 1000 µM) were obtained from single cultivations.


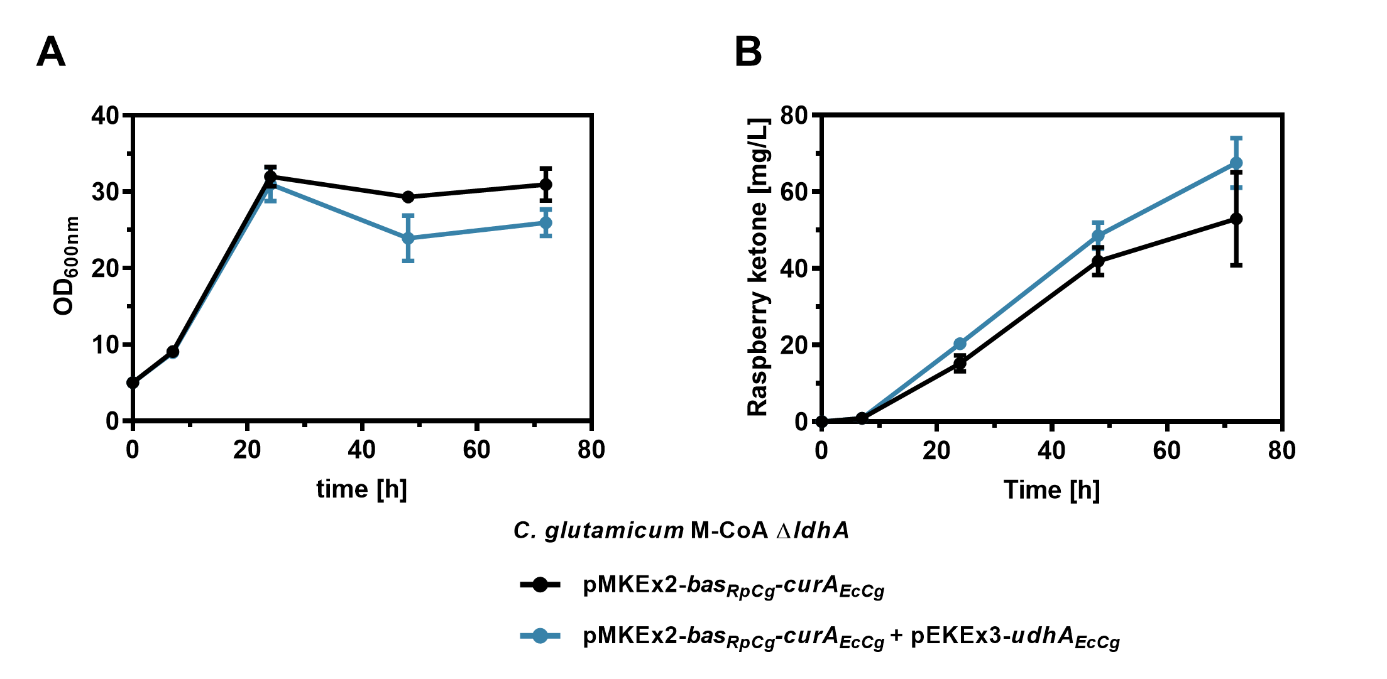


**Figure S4: Effect of heterologous expression of a codon-optimized *udhA_EcCg_* gene variant encoding the cytosolic transhydrogenase from *E. coli* on (A) growth of *C. glutamicum* and (B) raspberry ketone synthesis.** *C. glutamicum* M-CoA Δ*ldhA* harboring the indicated expression plasmids was cultivated in 50 mL CGXII medium with 4 % glucose and 500 mg/L supplemented *p*-hydroxybenzalacetone in baffled flasks at 30 °C and 130 rpm for 72 h. The depicted data represent mean values with standard deviations from biological triplicates.


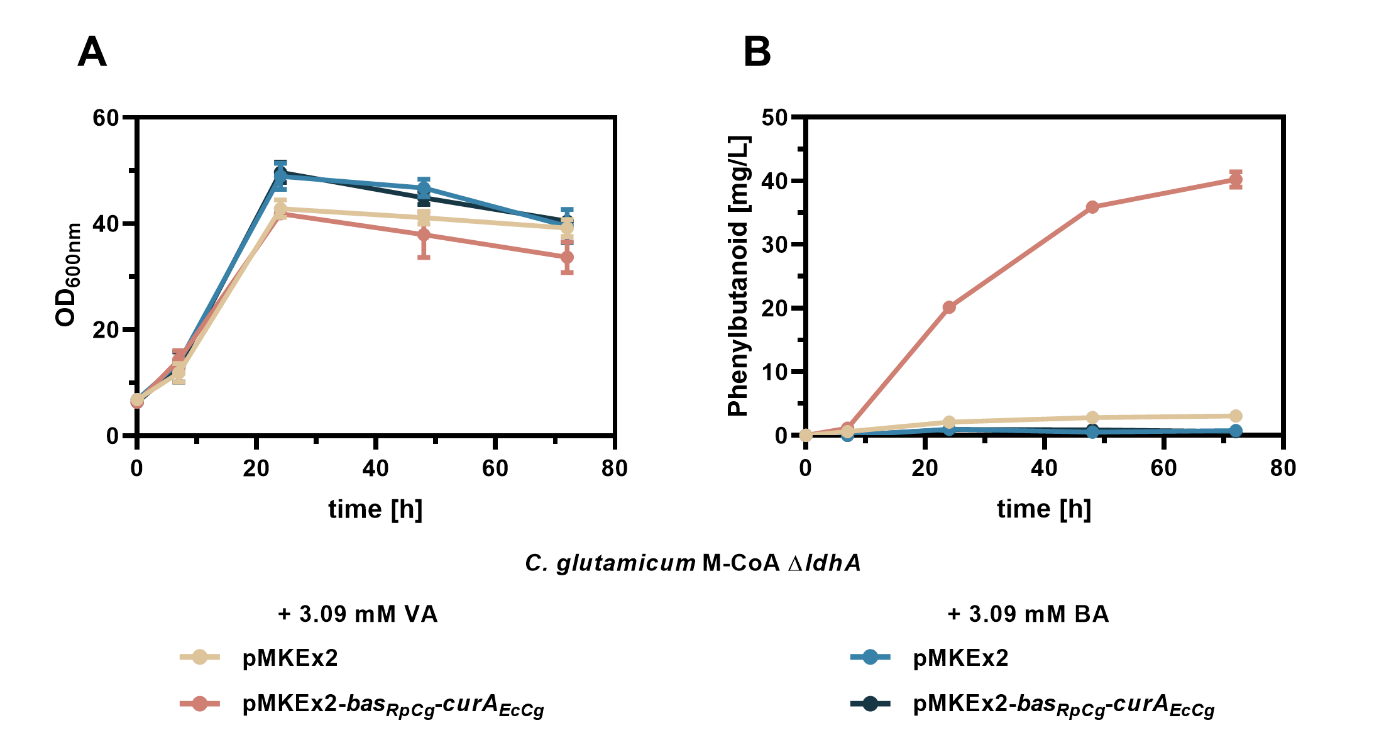


**Figure S5: Effect of *curA_EcCg_* expression on the reduction of diketide intermediates in *C. glutamicum*.** *C. glutamicum* M-CoA Δ*ldhA* optionally harboring one of the indicated expression plasmids was cultivated in 50 mL CGXII medium with 4 % glucose and 3.09 mM supplemented diketides vanillylidenacetone (VA) and benzalacetone (BA) in baffled flasks at 30 °C and 130 rpm for 72 h. Microbial growth (A) and phenylbutanoid synthesis (B) were followed over the process time. The depicted data represent mean values with standard deviations from biological triplicates.
